# CSF protein clearance impairment revealed using stable isotope kinetics in normal pressure hydrocephalus

**DOI:** 10.1093/braincomms/fcag029

**Published:** 2026-02-04

**Authors:** Claire A Leckey, Tatiana A Giovannucci, Eimear C Murphy, Eleanor M Moncur, Kanza Tariq, Aram Aslanyan, Michael Schöll, Meera Srikrishna, William Coath, Suzanne Barker, Dylan Esguerra, Ahmed K Toma, Laurence D Watkins, Lewis Thorne, Sylvain Lehmann, Jerome Vialaret, Selina Wray, Randall J Bateman, Kevin Mills, Donald L Elbert, Laura Pellegrini, Ross W Paterson

**Affiliations:** Department of Neurodegenerative Disease, UCL Queen Square Institute of Neurology, University College London, London WC1N 3BG, UK; Translational Mass Spectrometry Research Group, UCL Great Ormond Street Institute of Child Health, University College London, London WC1N 1EH, UK; Department of Neurodegenerative Disease, UCL Queen Square Institute of Neurology, University College London, London WC1N 3BG, UK; Translational Mass Spectrometry Research Group, UCL Great Ormond Street Institute of Child Health, University College London, London WC1N 1EH, UK; Department of Neurodegenerative Disease, UCL Queen Square Institute of Neurology, University College London, London WC1N 3BG, UK; Department of Neurosurgery, National Hospital for Neurology and Neurosurgery, University College Hospitals NHS Trust, London WC1N 3BG, UK; Department of Neurosurgery, National Hospital for Neurology and Neurosurgery, University College Hospitals NHS Trust, London WC1N 3BG, UK; Department of Neurodegenerative Disease, UCL Queen Square Institute of Neurology, University College London, London WC1N 3BG, UK; Department of Neurodegenerative Disease, UCL Queen Square Institute of Neurology, University College London, London WC1N 3BG, UK; Department of Psychiatry and Neurochemistry, University of Gothenburg, Gothenburg 413 45, Sweden; Department of Psychiatry and Neurochemistry, University of Gothenburg, Gothenburg 413 45, Sweden; Department of Neurodegenerative Disease, UCL Queen Square Institute of Neurology, University College London, London WC1N 3BG, UK; Department of Neurodegenerative Disease, UCL Queen Square Institute of Neurology, University College London, London WC1N 3BG, UK; Department of Neurology, School of Medicine, University of Washington, Seattle WA 98195, USA; Department of Neurosurgery, National Hospital for Neurology and Neurosurgery, University College Hospitals NHS Trust, London WC1N 3BG, UK; Department of Neurosurgery, National Hospital for Neurology and Neurosurgery, University College Hospitals NHS Trust, London WC1N 3BG, UK; Department of Neurosurgery, National Hospital for Neurology and Neurosurgery, University College Hospitals NHS Trust, London WC1N 3BG, UK; Institute for Regenerative Medicine and Biotherapy, Institute for Neurosciences of Montpellier, 34090, France; Institute for Regenerative Medicine and Biotherapy, Institute for Neurosciences of Montpellier, 34090, France; Department of Neurodegenerative Disease, UCL Queen Square Institute of Neurology, University College London, London WC1N 3BG, UK; Department of Neurology, Washington University St Louis, St Louis, MO 63110, USA; Translational Mass Spectrometry Research Group, UCL Great Ormond Street Institute of Child Health, University College London, London WC1N 1EH, UK; Department of Neurology, Washington University St Louis, St Louis, MO 63110, USA; Centre for Developmental Neurobiology, Kings College London, London SE1 1UL, UK; Department of Neurodegenerative Disease, UCL Queen Square Institute of Neurology, University College London, London WC1N 3BG, UK; UK Dementia Research Institute at UCL, University College London, London WC1E 6BT, UK

**Keywords:** neurosurgery, cognitive, dementia

## Abstract

Normal pressure hydrocephalus is a common cause of gait and cognitive impairment in older adults, marked by excessive CSF accumulation. Genetic studies suggest impaired fluid clearance, and clinical symptoms can improve after CSF diversion. However, no fluid biomarkers exist to explore CSF accumulation mechanisms, assist diagnosis, or predict response to treatment. Stable isotope labelling kinetics is a clinical research tool that uses non-radioactive isotopes to label newly translated proteins, enabling measurement of their appearance (synthesis) and disappearance (clearance) in compartments like CSF. This study aimed to develop a novel method to capture protein turnover in CSF and assess whether clearance disruption is evident in normal pressure hydrocephalus with extended follow-up. Proteins of interest were identified via mass spectrometry in human CSF and choroid plexus organoid-derived CSF-like fluid. Protein origin and synthesis rates were evaluated by labelling organoids with ^13^C_6_-leucine. Label incorporation was measured using targeted mass spectrometry to determine the ratio of labelled to unlabelled peptide. A proof-of-concept case-control study was then conducted in specialist neuroscience centres. Participants received intravenous ^13^C_6_-leucine and underwent serial CSF withdrawal via lumbar drain, with matched blood sampling for up to 72 h. Patients undergoing CSF drainage and controls were recruited sequentially. Targeted mass spectrometry was used to determine protein production and clearance rates. To determine the clinical relevance of these protein turnover rates to CSF flow, they were correlated with direct measurements of CSF production captured using a LiquoGuard machine linked to the lumbar CSF drain. We captured choroid plexus protein kinetics in human organoids and the CSF of participants undergoing CSF drainage (*n* = 10) or controls (ventricular CSF *n* = 4; lumbar CSF *n* = 5). The case and control cohorts varied in sex (NPH = 80% male and controls = 22% male) and in age. There was no significant age difference between NPH and the lumbar control cohort (*n* = 5) (NPH: 75 (71–78) versus 70 (63–84) years old; *P* = 0.2438). We found that transthyretin is abundantly secreted by choroid plexus organoids, and observed correlations with CSF transthyretin synthesis rates and volume of CSF production *in vivo* (*P* = 0.738; *P* < 0.05). Clearance rates of transthyretin are ∼10 fold slower in normal pressure hydrocephalus compared with controls, suggesting impaired CSF protein clearance. This method is a novel clinical tool for interrogating CSF protein dynamics and may have utility in tracking CSF flow clinically.

## Teaser

Leckey, Giovannucci *et al*. developed an assay to measure protein secretion and clearance from spinal fluid. They found that people with normal pressure hydrocephalus clear transthyretin more slowly than unaffected individuals, highlighting potential new diagnostic tools and therapeutic approaches.

## Graphical Abstract

**Graphical Abstract:**
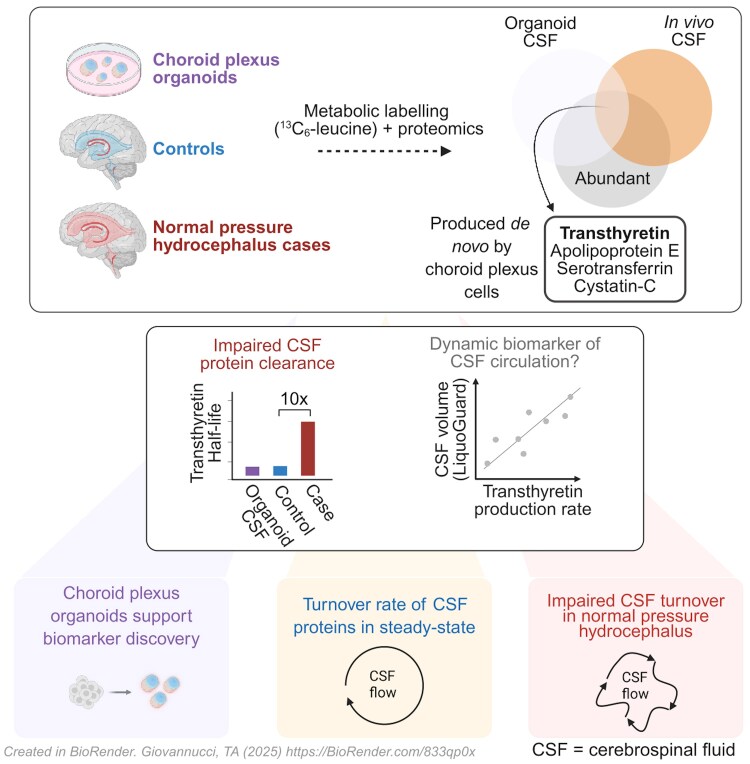

## Introduction

Normal pressure hydrocephalus (NPH) is a clinical syndrome characterized by excessive CSF accumulation resulting in ventriculomegaly, gait and cognitive impairment and urinary incontinence.^1^ It is common in the elderly, with an estimated prevalence of around 4% in those over 65 years.^2^ A radiological characteristic is a disproportionately enlarged subarachnoid space (DESH) on imaging.^3^

Little is known about the disease mechanism(s) of NPH, but individuals often respond clinically to CSF diversion through lumbar or ventriculoperitoneal shunting, and a growing body of genetic and functional imaging data supports the notion of it being a distinct disease entity, characterized by abnormal CSF circulation. Two genetic risk factors have been previously identified; SFMBT1,^4,5^ and CFAP43.^6^ Both implicate failure of CSF circulation, but it is not clear whether they impact CSF secretion or reabsorption. A recent GWAS study identified six novel risk genes significantly associated with NPH, several of which have been linked to the function of important fluid barriers in CSF circulation including the blood-brain and blood-CSF barriers.^7^ The only *in vivo* clinical study of CSF flow to date involved injected radio-opaque contrast into the intrathecal space and this demonstrated slower clearance in NPH compared with controls.^8^

CSF circulation occurs as part of normal brain physiology in humans, with proposed roles in nutrient provision, electrolyte homeostasis and waste clearance. The choroid plexus (ChP) has an important role in CSF production.^9^ It actively secretes and transports water, proteins and other metabolites across the blood-CSF barrier.^10^ It is unclear exactly how CSF and its constituents are cleared from the subarachnoid space, but reabsorption can occur through the arachnoid granulations, and other pathways including the meningeal lymphatics,^11^ and the glymphatic system.^12,13^ It is not clear how excessive CSF arises in NPH, but ChP CSF hypersecretion, ependymal denudation, and damage and scarring of intraventricular and parenchymal (glia–lymphatic) CSF pathways have been postulated based on animal studies and observations in humans with acquired hydrocephalus (e.g. post-infection or haemorrhage), with a prominent role of neuroinflammation.^12^

To date, the ability to investigate CSF circulation *in vivo* has been limited by a paucity of appropriate monitoring tools.^12^ Without *in vivo* biomarkers of CSF turnover it has been challenging to: (i) accurately diagnose NPH and identify individuals likely to respond to shunt; (ii) determine whether NPH is associated with dysregulation of CSF production, circulation or clearance; and (iii) develop and interpret static fluid biomarkers of NPH.

To address these challenges, we set out to develop a novel kinetic protein assay using the SILK technique,^14,15^ to measure the synthesis and clearance rates of ChP relevant targeted proteins in the CNS. The SILK method has been employed in the field of neurodegeneration to quantitate the production and clearance of key pathogenic proteins in Alzheimer's disease. We hypothesized that proteins abundantly expressed by the ChP could serve as biomarkers of ChP synthetic function. In addition, if these proteins do not easily cross the blood-CSF barrier, they could also serve as biomarkers of CSF circulation and clearance. In this study, we aimed to characterize and quantify a panel of ChP-associated proteins and measure synthesis and turnover rates in human stem-cell derived ChP organoids. We then aimed to do the same in humans, compare with organoids and correlate our findings with direct measures of CSF flow *in vivo*. To ensure the human subjects included had NPH, we monitored clinical response to CSF diversion and cognitive decline for up to 4 years and in two cases validated diagnosis with *ex vivo* brain biopsy.

## Materials and methods

### SILK in ChP organoids

#### Generation of ChP organoids

Human H1 ES cells were obtained from WiCell and used to generate ChP organoids using Stem Cell Technologies Cerebral Organoid kit (catalog nos. 08570 and 08571) reagents, as previously described.^16^ EBs were generated by seeding 4000 cells in a 96-well U bottom low attachment plate with EB media and 50 μM Y-27632 ROCK inhibitor for 3 days. On day 5, the culture media was switched to NI (neural induction) within the same 96-well plate. On day 7, EBs were embedded in 30 μL of Matrigel (Corning) using dimpled parafilm sheets, following the procedure as previously described,^17^ and incubated for 20 min at 37°C. Subsequently, the EBs was transferred to a 6-well plate with 3 mL of Expansion media in each well. For ChP patterning, 3 μM CHIR and 20 ng/mL BMP4 were added in Maturation media from day 10–17. From day 30, dissolved Matrigel (at a ratio of 1:50) was introduced to the Maturation media.

#### ChP profiling by single cell RNA sequencing

Organoid single-cell dissociation, library preparation and sequencing were previously described in Pellegrini *et al.*^16^ Briefly, single-cell dissociation was performed by pooling two organoids for each condition: 55-day H9 telencephalic organoids, 27-day H1 ChP (ChP sample 1), 46-day H1 ChP (ChP sample 2), and 53-day H1 ChP (ChP sample 3) into a 15 mL tube. Samples were incubated in 1 mL of Accumax (Sigma, A7089) with 400 μg DNase I and 15 μM actinomycin D at 37°C for 20 min with gentle agitation. At 5 min intervals, the tubes were flicked then pipetted 10 times. Clumps were allowed to settle and supernatant collected, to which 100 μL FBS was added before filtration on a 35 μm filter tube (Corning, 352235). Samples were spun at 300 *g* for 5 min. Dead Cell Removal kit and MACS column (Miltenyi, 130-090-101) were used to remove dead cells before another spin as previously described. Cells were resuspended in 0.04% BSA in PBS to load 16,000 cells per well on the 10X Chromium system (10X Genomics). Raw and processed human organoid sequencing data are available at the Gene Expression Omnibus (GEO) database GSE150903.

#### ChP organoid SILK

**Figure 1:**
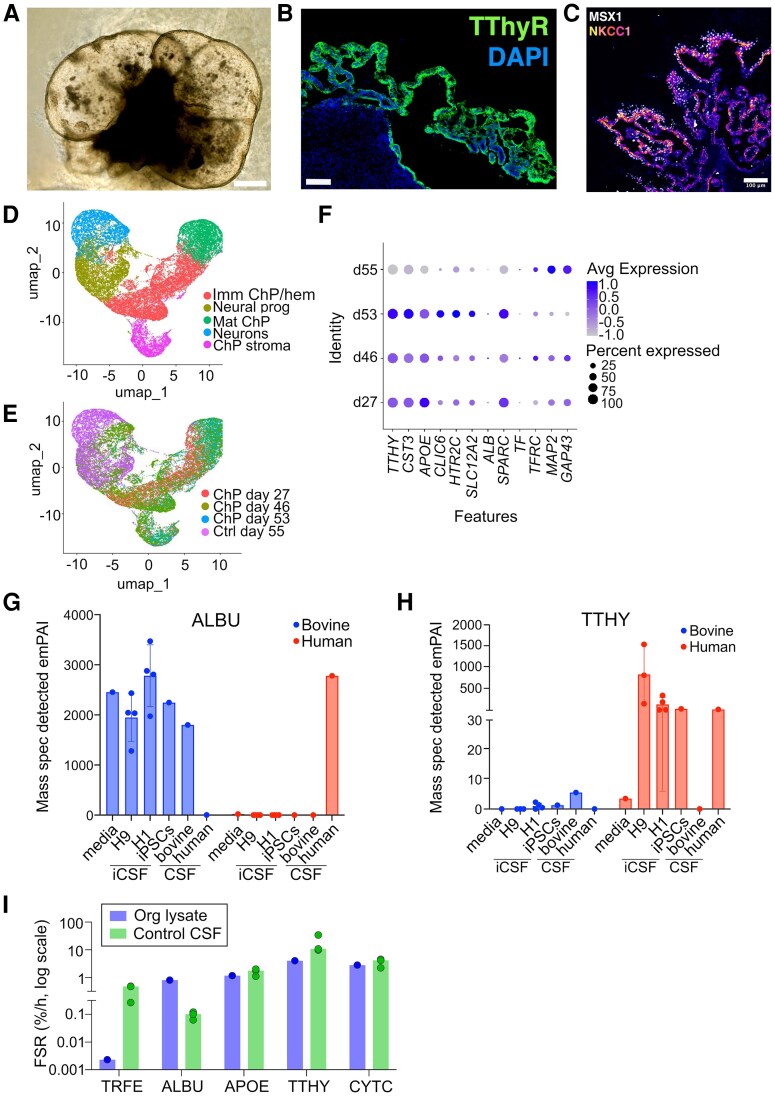
**Choroid plexus organoid characteristics.** (**A**) Bright field image of a H1 ChP organoid at day 42 that developed self-contained fluid compartments with CSF-like fluid. Scale bar, 250 μm. (**B**) Representative confocal image of whole ChP organoid from H1 at day 40 stained for TTHY in green and nuclear stain with DAPI in blue. Scale bar, 100 μm. (**C**) Representative confocal images of ChP epithelium from a H9 derived organoid at day 60 stained for ChP marker MSX1 and CSF transporter NKCC1, nuclei are stained with DAPI (blue). Scale bar, 100 μm. (**D**) Single Cell RNA (scRNA) sequencing of ChP organoids (ChP day27, ChP day46, ChP day53) and cortical organoid (Control day55) showing the different cell populations: ChP stroma, neurons, ChP epithelial cells (Mat ChP), hem/ChP progenitors (Imm ChP/hem) and neuronal progenitors (Neural prog). (**E**) scRNA sequencing showing different timepoints analysed (ChP day27, ChP day46, ChP day53, Ctrl day55). (**F**) Dot plot showing average expression and percentage of cells expressing the displayed enriched genes identified by scRNA sequencing (scRNA seq data from Pellegrini *et al.*^16^). *n* = number of cells; *n* = 5347 cells (ChP day27), *n* = 8603 cells (ChP day46), *n* = 8573 cells (ChP day53) and *n* = 10 327 cells (Control day55). (**G, H**) Bar charts representing the mean ± SD with scatter plots of mass spectrometry detected emPAI values for ALBU and TTHY peptides of human and bovine origin. Each datapoint represents samples from organoid media (*n* = 1), organoid CSF from 3 independent cell lines (human embryonic lines H9, *n* = 4; H1, *n* = 4 and iPSC line IMR90-4, *n* = 1), bovine foetal CSF (*n* = 1) and human adult CSF (*n* = 1; mass spectrometry data from Pellegrini *et al.*^16^). (**I**) Bar chart comparing median FSR (% protein produced/hour; shown in a log10 scale) of choroid plexus proteins synthesized by organoid (blue) labelled by SILK, and human control cerebrospinal fluid labelled by SILK (green). Each datapoint represents the FSR calculated from data pooled from two independent organoid batches (*n* = 2) or the FSR from an individual subject (*n* = 3), respectively. ALBU = albumin; APOE = Apolipoprotein E; ChP = Choroid Plexus; CYTC = Cystatin-C; CSF = cerebrospinal fluid; FSR = Fractional Synthesis Rate; NKCC1 = Na+/K+/2Cl− cotransporter; iCSF = organoid CSF; NPH = Normal Pressure Hydrocephalus; Org = organoid; TRFE = Serotransferrin; TTHY = Transthyretin.

ChP organoids were labelled with 50% mol [100% tracer to tracee ratio (TTR)] ^13^C_6_-leucine for one week and subsequently cultured in unlabelled media for 24 h (Fig. 1A). Organoids were then washed with fresh, unlabelled media and incubated with regular Maturation media for the 24 h chase. CSF-like organoid fluid was collected at 1, 6 and 24 h. For the organoid CSF collection: media was removed, organoids were washed with fresh media, and CSF extracted using a pulled glass microcapillary as previously described.^16^ Briefly, iCSF was collected using suction via a pulled glass microcapillary attached to filter and tubing. iCSF was centrifuged at 10 000 *g* for 10 min to pellet debris before supernatant was collected, snap frozen in liquid nitrogen and stored at −80°C. iCSF samples were prepared using the same protocol as CSF described below.

### ChP SILK in human subjects

#### Recruitment and SILK protocol for NPH participants (NPH cohort)

Individuals with suspected idiopathic NPH (iNPH) were recruited from the specialist hydrocephalus service at the National Hospital for Neurology and Neurosurgery, Queen Square, London between 2022 and 2023. Informed written consent was given. Individuals were prospectively assessed to have clinical and radiological features that met international criteria for probable NPH,^18^ with DESH. They were each examined by a board-certified neurologist and determined not to have features of a congenital disorder known to cause hydrocephalus nor an alternative neurodegenerative disease such as progressive supranuclear palsy (PSP). Specific symptoms and examination features were recorded according to a pre-specified questionnaire. A collateral history was obtained where possible and a timed walk was carried out by a member of their clinical team. Sample size was pragmatically determined based on previous pilot SILK studies.

Individuals then underwent SILK as previously described.^14^ Briefly, participants were labelled intravenously with ^13^C_6_-leucine at a concentration of 8.5 mg/mL prior to diagnostic lumbar drainage, with leucine administered at 3 mg/kg/h for 10 min, followed by 2 mg/kg/h for up to 9 h. The infusion was continued until drain insertion, thus infusion duration varied depending on clinical factors which influenced the time of surgery.

To correct for variation in infusion duration and other physiological factors that might influence leucine metabolism, TTRs were adjusted for infusion duration using plasma leucine enrichment at plateau, measured using HILIC-MS/MS. Research CSF was removed regularly for the duration of the drainage period. CSF was collected in a 10 mL syringe and transferred to a polypropylene Falcon container and centrifuged at 4°C for 10 min at 1500 *g*. Samples were then aliquoted into 1.5 mL microcentrifuge tubes and stored at −80°C prior to UPLC-MS/MS.

A sub-cohort consented to ex vivo brain biopsy collection during subsequent ventriculoperitoneal shunt insertion. Full thickness parietal biopsy was flash frozen on dry ice, and LCO and AT8 stained sections were visualized using fluorescence microscopy.

#### Recruitment and SILK protocol for control participants [SAH cohort; ‘Control (V)’]

Controls were patients with non-traumatic subarachnoid hemorrhage (SAH) at the Center Hospital Montpellier, who had recovered from their acute illness and CSF flow rate had plateaued to normal clinical parameters as determined by their clinicians. Their ventricular shunts remained for an additional 72 h to facilitate SILK. Leucine was administered at a rate of 3 mg/kg/h for 10 min, followed by 2 mg/kg/h for up to 9 h. CSF was withdrawn at regular intervals and processed prior to storage at −80°C as described above. Importantly these individuals did not have a history of a neurodegenerative disease and given their resolving acute illness were for the purposes of this study, setting aside their obvious limitations, were considered as healthy controls

#### Recruitment and SILK protocol for lumbar control participants [Amyloid negative cohort; ‘Control (L)’]

Controls were research volunteers, who had a lumbar drain placed at Washington University in St. Louis (WashU), as previously described.^14,19^ Leucine was administered and CSF was withdrawn and processed as described above.

#### Clinical measurement of CSF flow in NPH subjects

Lumbar drain (LD; Medtronic® Duet epidural catheter, USA) was inserted in-between the L3/L4 spinal vertebrae. LiquoGuard7 (MÖLLER Medical GmbH, Germany) was attached to the drainage catheters and the LiquoGuard7 external drainage tubing was primed using sterile saline to prevent CSF wastage.

The external intracranial pressure (ICP) transducer of the LiquoGuard7 pump was applied on patient's bodies in line with the external auditory meatus. Participants were lying flat for the procedure, which included an initial 20 min for CSF flow rates to stabilize and an additional 30 min to calculate CSF production rate. The LiquoGuard7 parameters brought the ICP pressure to 0 mmHg to allow CSF to drain freely into the LiquoGuard7 drainage bag. The LiquoGuard7 software was used to analyse flow rate data and to calculate an hourly CSF production rate (mL/h).

#### Neuroimaging of NPH subjects

Participants had brain imaging (either MRI or CT) as part of their normal clinical care, either to confirm diagnosis or for monitoring. Using a deep-learning-driven CT-brain quantification pipeline,^20^ 10 pre- and post-shunt CT scans from five unique participants from the NPH cohort underwent brain segmentation analysis. This pipeline enables an automated quantitative segmentation of brain structures, including VCSF and ICV in brain CT images. Employing trained deep-learning models, the pipeline efficiently segments various tissue classes within the input CT images. Subsequently, segmented VCSF and ICV maps are binarized, and their volumes are extracted. From these volumes, an iNPH-related CT-based volumetric measure, VCSF/ICV, analogous to the 3D version of Evans’ Index, was derived. Further, changes in CT-VCSF volume and CT-VCSF/ICV are visualized pre- and post-shunt, and over days after shunt.

#### Measurement of plasma ^13^C_6_-leucine enrichment by HILIC-MS/MS

To quantitate labelled leucine enrichment in plasma, a hydrophilic interaction liquid chromatography–tandem mass spectrometry (HILIC-MS/MS) assay was adapted from Prinsen *et al.*^21^ to measure ^13^C_6_-/^12^C_6_-leucine ratios, with the following optimisations. Analysis was performed using an Acquity H-Class Ultra Performance Liquid Chromatography (UPLC) system, with an ACQUITY UPLC BEH Amide column (100A; 1.7 µm, 2.1×50 mm) attached to a VanGuard UPLC BEH Amide precolumn (2.1×5 mm), which was coupled to a Xevo TQ-S triple quadrupole mass spectrometer operated in positive electrospray ionisation (ESI^+^) mode (Waters, UK). Chromatographic separation was performed over a 10-min HILIC gradient using mobile phases A (10 mM ammonium formate in 85% ACN 0.15% FA) and B (10 mM ammonium formate in ultrapure Milli-Q water 0.15% FA). The column was primed and equilibrated for 45 min in initial conditions (100% A at 0.4 mL/min) and kept at 35°C. At injection (1 μL/sample), the column was kept in initial conditions for 3 min until a linear gradient of increasing %B began. From 3 to 3.1 min B was increased to 5.9%, and from 3.1 to 5 min was increased to 17.6%, followed by a final increase to 29.4% from 5 to 6 min. The flow rate was then increased to 0.6 mL/min and the column re-equilibrated in 100% A for 4 min. Mass spectrometer parameters were as follows: source temperature (150°C), capillary voltage (1.00 kV), desolvation temperature (550°C), desolvation gas flow (1000 L/h) and cone gas flow (150 L/h).

Ion transitions for ^12^C_6_-leucine (*precursor: 132.102 m/z, product: 86.100 m/z*), ^13^C_6_-leucine (*precursor: 138.122 m/z, product: 91.139 m/z*) and ^13^C_6_-,^15^N_2_-lysine internal standard (*precursor: 155.127 m/z, product: 90.100 m/z*) were analysed by multiple reaction monitoring (MRM) and acquired data imported into Skyline (MacCoss Lab, Seattle, USA) for processing and peak integration. Peak areas were exported into Microsoft Excel and leucine TTR calculated as molar ^13^C_6_-leucine/^12^C_6_-leucine peak area ratios.

#### UPLC-MS/MS analysis of ChP peptides in iCSF and CSF

NPH and control SAH CSF samples were thawed on ice before 350 µL of CSF was spiked with 150 ng of yeast enolase internal standard (E6126, Sigma-Aldrich). Protein was precipitated by adding 3-sample volumes of ice-cold acetone, which was incubated at −20°C overnight (16 h). Samples were centrifuged at 16 000 *g* for 20 min at 4°C, the acetone supernatant removed and the protein pellet allowed to air dry. The pellet was resolubilised in digest buffer (6 M urea, 2 M thiourea, 2% ASB-14, 200 mM Tris-HCl, pH 8) for at least 20 min before reduction and alkylation of peptides by incubation with 1,4-dithioerythritol (DTT; Sigma-Aldrich, UK) and iodoacetamide (IAA; Sigma-Aldrich, UK) respectively. 2 µg of Trypsin-LysC (MS grade, Promega) was added to each sample followed by incubation at 37°C overnight (16 h). Tryptic peptides were purified by C_18_ solid-phase extraction (Bond Elute, Agilent Technologies, UK). Purified peptides were lyophilized using a centrifugal evaporator (SpeedVac, Eppendorf) and reconstituted in 50 µL 3% acetonitrile (ACN) 0.1% formic acid (FA) prior to UPLC-MS/MS analysis.

Targeted analysis of the ChP protein SILK panel *in vitro* and *in vivo* was performed by tandem MS on an Acquity I-Class PLUS UPLC system coupled to a Xevo TQ-XS mass spectrometer operated in positive electrospray ionisation (ESI+) mode (all Waters, UK). A multiplexed assay was developed to monitor peptides that were proteotypic for human transthyretin (TTHY), cystatin-c, apolipoprotein E, albumin and serotransferrin using peptide standards (GenScript, UK). Peptide sequences and their respective ion transitions monitored in the ChP SILK assay for unlabelled and labelled proteins are provided in Supplementary Table 3.

Samples were injected (0.5 µL) onto an Acquity Premier peptide BEH C18 column (300 Å, 1.7 μm, 2.1× 50 mm) held at 50°C and peptides were separated over a 16-min reverse-phase UPLC gradient as detailed in Leckey *et al*.^22^

Acquired data was imported into Skyline for peak picking and integration. Peak areas were exported as a .csv file for further analysis.

#### Compartmental modelling of lumbar versus ventricular CSF

To determine whether differences in clearance rates between NPH and SAH-control participants could be attributed to CSF compartmental differences (lumbar versus ventricular sampling), lumbar CSF SILK data previously studied at WashU [Control (L) cohort] was compared with ventricular CSF SILK data [Control (V) cohort] for TTHY during modelling. The Control (L) cohort included cognitively normal individuals who were amyloid negative on amyloid PET or CSF amyloid beta (Aβ) Aβ42/40 ratio. A detailed SILK protocol for these five subjects has been previously described.^14^ Study participants were administered an initial bolus of ^13^C_6_-leucine for 10 min of 3 mg/kg prior to continuous intravenous infusion at a rate of 1.8–2.5 mg/kg/hr for up to 9 h. CSF and plasma samples were collected at 1- or 2-h intervals over 36 h and enrichment of ^13^C_6_-leucine was quantified using capillary gas-chromatography MS (GC-MS). SILK data for the validation control subjects was acquired by untargeted high-resolution MS at Université de Montpellier using the SILAV approach as previously described.^23^

### Statistical analysis

#### Quantitation of ChP kinetic rates in vitro and in vivo

The fractional synthesis rate (FSR) of each protein was calculated from the linear regression of the upslope during the chase divided by the mole fraction labelled leucine (^13^C_6_-leucine) at plateau in plasma during the pulse. Due to the unpredictable timing of theatre slots for NPH participants, pulse duration varied and thus FSRs for the NPH cohort were calculated using the plasma leucine TTR from a subject who reached a steady plateau of the tracer during labelling. Fractional clearance rate (FCR) was derived from the negative slope of the natural logarithm of the clearance portion of the kinetic curve (from peak of labelling onwards in vivo; using all timepoints during the ‘chase’ *in vitro*). FCRs were captured in 8/10 NPH participants due to differences in chase periods between participants.

The FSR was calculated using the standard formula:


FSR=(Et2−Et1)NfL/(t2−t1)/PrecursorE

where (E*t*2−E*t*1)NfL/(*t*2−*t*1) was defined as the slope of the linear regression during the labelling (‘pulse’) divided by the leucine enrichment in plasma in vivo as determined by HILIC-MS/MS (in vitro, where the ^13^C_6_-leucine enrichment in media was equal to the quantity of ^12^C-leucine, this equals 1). FSRs were captured in 3/4 Control (V) participants due to unavailability of leucine enrichment in plasma from one of them.

Protein half-life was calculated using the exponential decay equation *t* 1/2 = ln(2)/*k*, where *k* is calculated from the non-linear, exponential regression of the clearance portion of the kinetic curve.

To determine if FSR and FCR measures were significantly different between controls and NPH participants, Mann-Whitney U-tests were performed in IBM SPSS Statistics (version 27, IBM). Figures displaying the labelled/unlabelled peptide percentages over time were generated using Python.

### Statistics

All statistical analyses were performed using Excel, Prism v.9.5.1 (GraphPad Software) and IBM SPSS Statistics (version 27, IBM).

To assess data normality, Shapiro-Wilk tests were performed. Spearman's correlation analyses assessed the association between CSF production rate measured in-clinic by LiquoGuard7 and FSR of TTHY by SILK *in vivo*. Unpaired *t*-test was performed to assess if there were significant differences in TTHY half-life between NPH patients and controls. *P* ≤ 0.05 was considered statistically significant.

## Results

### Choroid plexus organoid characteristics

Human choroid plexus (ChP) organoids form fluid-filled sacks that contain clear colourless fluid by day 42 (Fig. 1A). We have previously shown that human *in vitro* ChP organoid fluid (‘iCSF’) has a CSF protein profile similar to human CSF.^16^ The epithelial cells present in the organoid form a polarized barrier, with tight junctions on the apical side and specialized ChP transporters regulating entry of molecules across the barrier. Staining of ChP and CSF transporter markers show that organoids display characteristics of ChP cells by day 40 *in vitro* (Fig. 1B and C). Previously published single-cell RNA sequencing of ChP organoids show development and maturation of distinct cell populations including ChP epithelial and stromal cells, as well as some neuronal progenitors and neurons.^16^ Single-cell RNA sequencing between day 27 and day 53 show the enrichment of the ChP epithelial and stromal cells in ChP organoids compared with control, unguided cerebral organoids (Fig. 1D and E). We also show expression of albumin, cystatin-C (CYTC) and transthyretin (TTHY), indicating abundant expression of CYTC and TTHY and very little expression of albumin (Fig. 1F). We selected a panel of proteins that were highly abundant in both iCSF and human control CSF, known to be translated by, or transported across ChP epithelial cells, and that were also amenable to SILK (contain leucine residues). The proteins selected were albumin, apolipoprotein E (APOE), CYTC, serotransferrin and TTHY. Since some of these proteins are also constituents of organoid development media, we used differences in sequence homology with targeted proteomics to differentiate human from bovine origin protein (Fig. 1G and H). Albumin peptides in iCSF consisted mostly of bovine albumin, indicating that albumin was transported from the media across the media/CSF barrier. TTHY in iCSF was almost exclusively human indicating that it was translated by organoid cells.

### ChP organoid SILK

Using SILK proteomics and plotting the tracer-to-tracee ratio (TTR) of peptides corresponding to the ChP-related proteins of interest, we captured the turnover of all five proteins in ChP organoid fluid including: plasma derived proteins, albumin and serotransferrin, and ChP derived proteins: TTHY, CYTC and APOE (Supplementary Fig. 1).

The rate of labelling of plasma derived proteins was very low with an albumin TTR of ∼1.5% (FSR 0.004%/h), and serotransferrin TTR of <0.4% (FSR −0.01%/h) indicating limited to no labelling in organoid fluid during the first 24-h pulse phase, and thus very low synthesis. However, the TTR of albumin in organoid lysate was ∼10% (FSR 0.8%/h) and for serotransferrin 0.15% (FSR 0.002%/h) suggesting some albumin is synthesized by ChP organoid cells during the pulse phase whilst there is negligible labelling of serotransferrin, excluding its synthesis by ChP (Fig. 1I and Supplementary Fig. 2A and B). By contrast, the TTR of peptides corresponding to ChP derived proteins was around 20–50 fold higher, depending on the protein (Fig. 1I and Supplementary Fig. 2C–E): TTHY (iCSF peak-of-labelling TTR ∼50%; FSR 4.51%/h; organoid lysate peak TTR 50%; FSR 4.0%/h), CYTC (iCSF peak TTR ∼60%; FSR 5.78%/h; organoid lysate peak TTR ∼50%; FSR 2.8%/h) and APOE (iCSF peak TTR 35%; FSR 2.20%/h; organoid lysate TTR 20%; FSR 1.2%/h).

The FCR of all proteins was extremely low (<0.1%/h) in organoid CSF and lysate (Supplementary Figs. 1G–K and 2F–J), indicating that no significant protein clearance occurs in either compartment during the time window studied.

### Human subject characteristics

**Figure 2:**
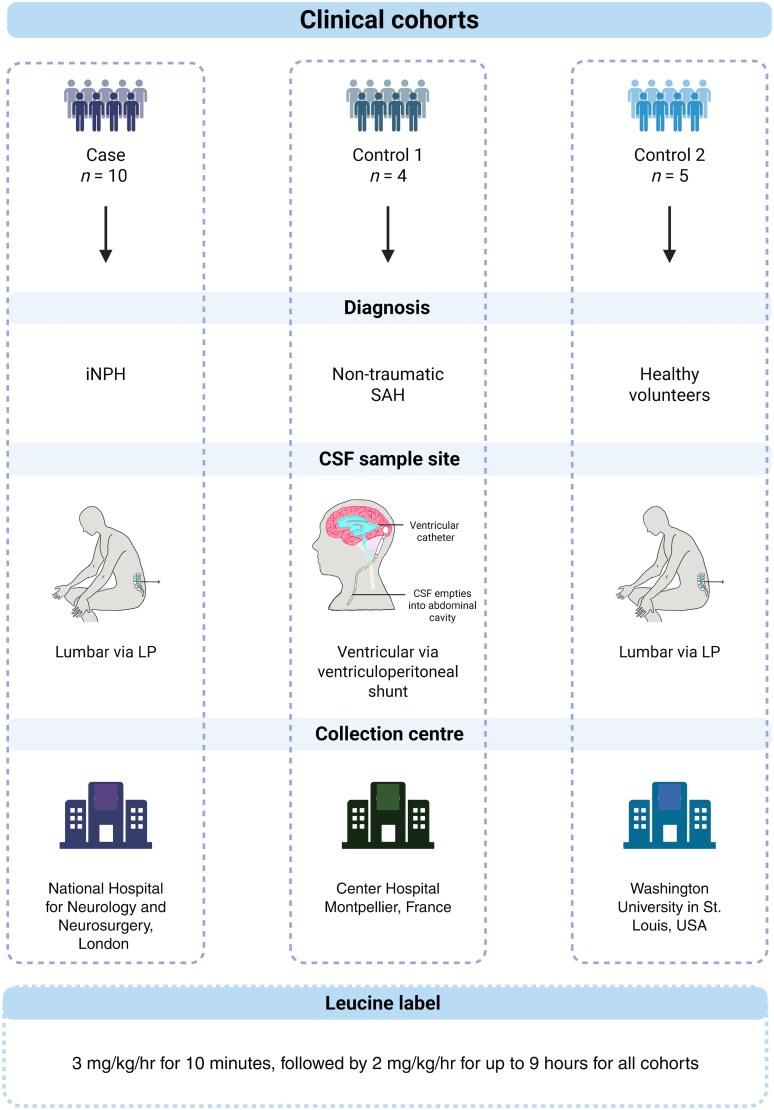
**Schematic overview of study participants.** The clinical cohorts consisted of the NPH cohort and two control cohorts including the SAH cohort (‘Control (V)’) and the amyloid negative cohort (‘Control (L)’). CSF = cerebrospinal fluid; L = lumbar; NPH = Normal Pressure Hydrocephalus; SAH = Subarachnoid Haemorrhage; V = ventricular. Figure 2 created in BioRender. Murphy, E. (2026) https://BioRender.com/1ibmp0q.

Individuals were recruited to measure protein kinetics *in vivo* using SILK. Subject characteristics are summarized in Supplementary Table 1. In total, nineteen individuals were included in this study (Fig. 2); 10 individuals with suspected NPH, four individuals recovered from the acute phase of SAH (‘SAH-controls’) where ventricular CSF was collected (Control (V)), and five controls from a validation cohort; where lumbar CSF was collected. This cohort was composed of five amyloid negative age matched controls (Control (L)).

There was no significant age difference between the NPH and lumbar control cohorts [NPH: 75 (71–78) versus 70 (63–84) years old; *P* = 0.2438]. Comparing the NPH and SAH-control groups, the controls were younger [NPH: 75, (71–78) versus 57 (46–65) years old; *P* = 0.0012]. The sex distribution of the NPH and SAH-control groups also differed. The NPH cohort had abnormal gait on examination and the majority of individuals reported urinary incontinence (80%) (Supplementary Table 1). Most individuals with NPH reported cognitive symptoms; 70% reported subjective impairment of episodic memory and 50% problems with executive function. Objective cognitive screening showed cognitive impairment to be mild (MMSE: median 28; IQR 26–28; *n* = 9). None had CSF biomarker support for amyloidosis, measured using CSF Aβ42/40 ratio. All individuals had imaging features of disproportionately enlarged subarachnoid-space hydrocephalus, determined by a neuroradiologist. 5/10 had CT imaging before and after shunting, and demonstrated reversibility of ventriculomegaly following shunt insertion with a mean change in ventricular volume of −27.5 mL; −13.11% change (Supplementary Fig. 3). Seven suspected NPH patients responded to CSF diversion with an improvement in gait and/or cognition. Two did not clinically improve and one participant died of other unrelated causes and the surgical outcome could not be determined. Two individuals had ex-vivo brain biopsies collected during ventriculoperitoneal shunt insertion, and did not have amyloid plaques nor tau tangles.

We followed participants up for a median of 31 months (range 17–48 months) and found that 8/9 had a sustained improvement in gait and 7/9 reported no further cognitive decline.

### In vivo human ChP SILK

**Figure 3:**
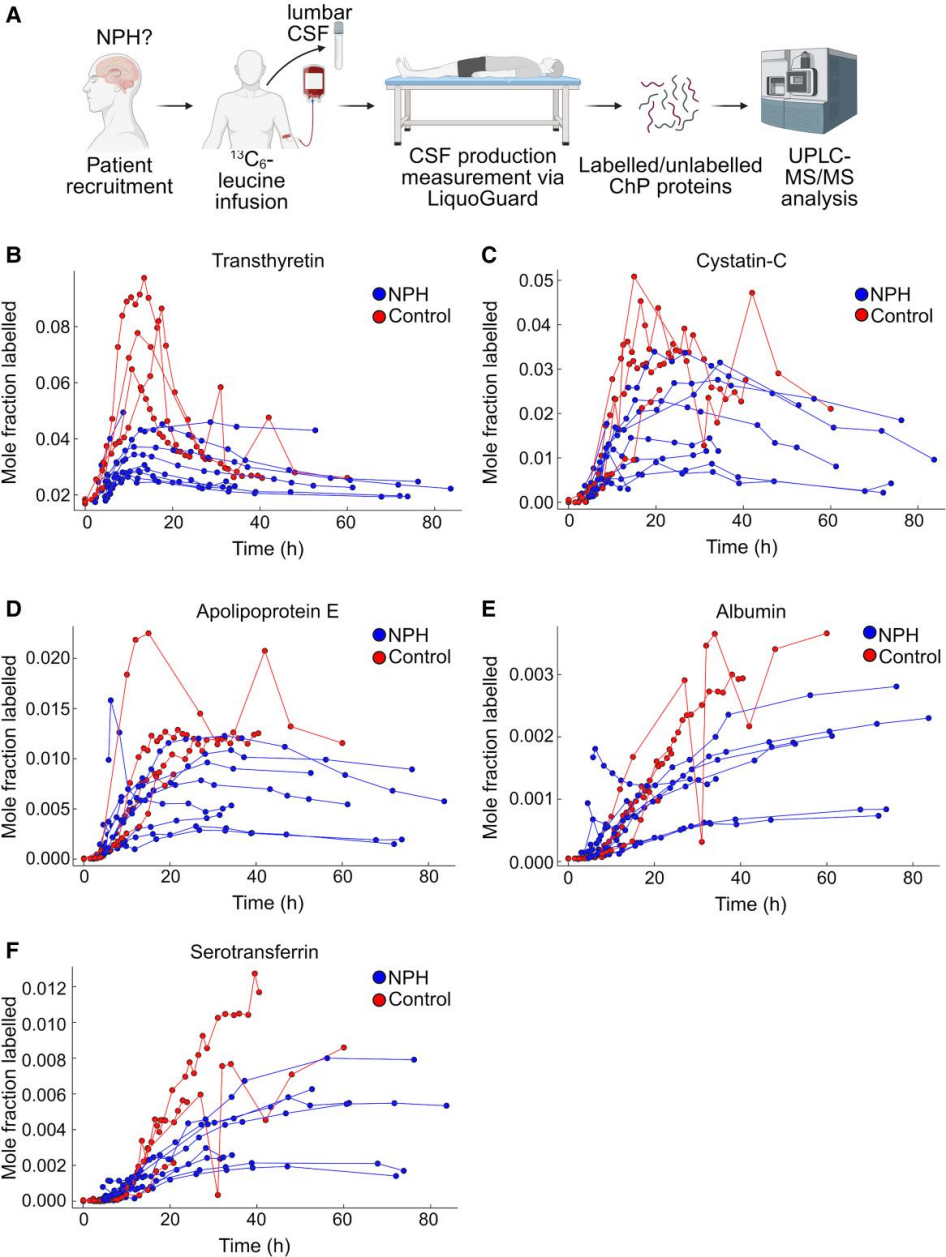
**Choroid plexus protein turnover in human CSF: plasma and ChP derived proteins.** (**A**) Schematic of SILK labelling method followed on suspected NPH patients. (**B–F**) *In vivo* SILK time course profiles of ChP proteins in both NPH (blue curves; *n* = 10) and control (SAH cohort) CSF (red curves; *n* = 4) for (**B**) Transthyretin (ALG peptide), (**C**) Cystatin-C, (**D**) Apolipoprotein-E, (**E**) Albumin, (**F**) Serotransferrin. Each dot represents the measured mole fraction labelled at a given timepoint. This is a measure of the amount of labelled peptide over the total peptide pool. ChP = Choroid Plexus; CSF = cerebrospinal fluid; NPH = Normal Pressure Hydrocephalus; UPLC-MS/MS = Ultraperformance Liquid Chromatography coupled to tandem mass spectrometry. Figure 3A created in BioRender. Murphy, E. (2026) https://BioRender.com/h49s738.

The workflow of ChP SILK *in vivo* is summarized in Fig. 3A. In the time window studied (72 h), we captured the FSR and FCR rates of TTHY, CYTC and APOE, as shown in Fig. 3B–D. TTHY had the highest FSR, followed by CYTC and APOE (Supplementary Table 2). Serotransferrin and albumin had much lower FSR, which continued to rise at 72 h, thus only synthesis rates could be measured (Fig. 3E and F). FSR was significantly higher in controls compared with NPH for both the ALG and TSE peptide monitored for TTHY (*P* < 0.05) and serotransferrin (*P <* 0.05).

**Figure 4:**
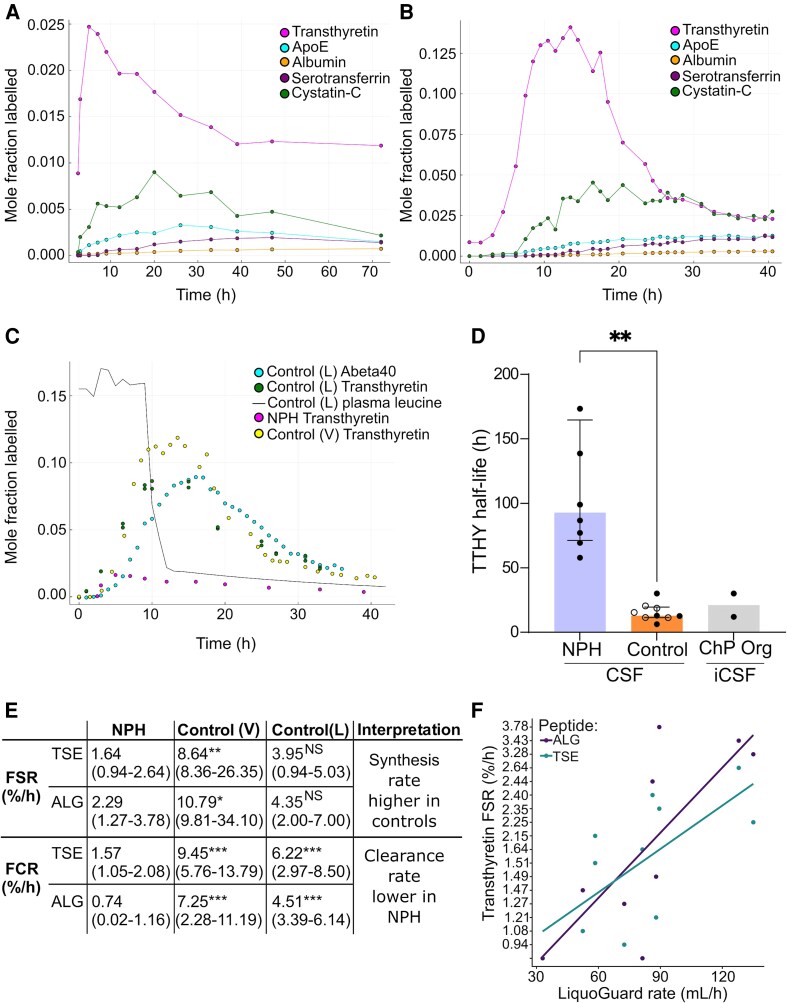
**Summary of ChP kinetics *in vivo*.** (**A**) Kinetic curve of five peptides within a single subject (NPH group). (**B**) Kinetic curve of five peptides within a single subject (control group). (**C**) Comparison of ChP kinetics in ventricular and lumbar CSF. Control (L): data from one individual from the lumbar control cohort; Control (V): data from one individual from the ventricular control cohort. (**D**) TTHY turnover in NPH patients (*n* = 8), Controls [Control (V) and Control (L)] (*n* = 9) and ChP organoids (*n* = 2). Bars represent the median +/− interquartile range. Control (L) are depicted with circles. Control (V) are depicted with filled black circles. *t*(7.165) = 4.733, *P* = 0.0020. ** Represents significance at *P* < 0.0021 (unpaired *t*-test with Welch's correction). (**E**) TTHY TSE peptide FSR and FCR summary *in vivo;* median (range). Mann-Whitney U test was performed to evaluate whether TTHY synthesis (FSR) and clearance (FCR) rates differed by group. The results indicated that the NPH cohort had higher synthesis rates than Control (V) (ALG: *U* = 0, *P* = 0.012; TSE: *U* = 0, *P* = 0.009) and lower clearance rates (ALG: *U* = 0, *P* = 0.004; TSE: *U* = 0, *P* = 0.004). The NPH group also had lower clearance rates than Control (L) (ALG: *U* = 0, *P* = 0.002; TSE: *U* = 0, *P* = 0.004). The synthesis rates were not significantly different between NPH and Control (L) (ALG: *U* = 8, *P* = 0.093; TSE: *U* = 8, *P* = 0.060). Significance between NPH and each control group is represented as follows: *P < 0.05; **P < 0.01; ***P < 0.005; NS = Not significant. (**F**) Spearman's correlation analyses assessed the association between CSF production rate measured in-clinic by LiquoGuard7 and the FSR of TTHY peptides ALG (*P* = 0.037, *ρ* = 0.738, *R*^2^ = 0.483) and TSE (*P* = 0.042, *ρ* = 0.683, *R*^2^ = 0.519). ApoE = Apolipoprotein E; ChP = Choroid Plexus; CSF = cerebrospinal fluid; FSR = Fractional Synthesis Rate; FCR = Fractional Clearance Rate; iCSF = organoid CSF; L = Lumbar; NPH = Normal Pressure Hydrocephalus; Org = Organoid; TTHY = Transthyretin; V = Ventricular.

FCR was captured for TTHY, CYTC and APOE. For TTHY ALG peptide, FCR was ∼10-fold lower in NPH than controls (ALG peptide: *P* < 0.01; TSE peptide: *P* < 0.01). For comparison, the kinetic curves of all five proteins within single NPH and control subjects are shown in Fig. 4A and B.

### Comparing ChP kinetics in ventricular and lumbar CSF

Since SAH-control CSF was ventricular and NPH CSF lumbar, we investigated whether the site of CSF collection could confound SILK measurements. We compared FSR and FCR values for TTHY between the SAH controls (yellow-*ventricular*) and a validation control group ‘lumbar controls’ who were amyloid negative age-matched controls who donated lumbar CSF (dark green-*lumbar*), and showed strong alignment of their kinetic curves despite their CSF compartmental differences (single subject shown in Fig. 4C and other individuals in Supplementary Fig. 4). This makes it unlikely that differences found in controls versus NPH could be due to differences in kinetics between the two compartments and supports a defect in TTHY clearance in NPH that results in a longer TTHY half-life in CSF (Fig. 4D and E). It also provides confidence that this clinical SILK method is broadly reproducible between clinical centres. Although the analytical approaches at each SILK site utilized MS, direct comparison between the NPH and validation controls was not performed due to slight method variations across sites. However, an exploratory analysis comparing TTHY FCR between the two groups showed a lower TTHY FCR in NPH compared with controls in lumbar CSF (Supplementary Table 2). We also note that TTHY FSR was higher in ventricular controls compared with lumbar controls.

### Relating protein kinetics to measured CSF flow

In NPH subjects, TTHY FSR for both peptides monitored was positively and significantly correlated with CSF production as measured by LiquoGuard7 (*P* = 0.037, *ρ* = 0.738 for ALG peptide and *P* = 0.042, *ρ* = 0.683 for TSE peptide) (Fig. 4F). No association was observed between the rate of CSF production and the FSRs of CYTC, serotransferrin, albumin and APOE (*P* values = 0.5518, 0.8737, 0.8024 and 0.4194, respectively). This suggests that the FSR of TTHY in CSF could be a biomarker reflective of CSF production.

## Discussion

In this exploratory study we introduce a novel SILK method to measure the synthesis and clearance of ChP proteins in human CSF, revealing significant impairment in CSF protein clearance in NPH compared with controls. We also observed disruption of ChP synthetic function suggesting a possible feedback loop that may regulate CSF protein synthesis in humans. We speculate that ChP protein turnover may be a biomarker reflective of CSF bulk flow turnover. Our well-characterized clinical cohort, two with *ex vivo* brain biopsy, are followed for up to 4 years, providing confidence that they respond to CSF diversion, and most do not clinically manifest alternative neurodegenerative diseases.

Since there is no pathological hallmark of NPH,^24^ and still doubt that NPH is a distinct disease entity,^25^ we set out to determine if we could detect objective differences in protein kinetics in this population that might reflect CSF flow. We designed a panel of novel kinetic protein targets to enable us to track (i) protein synthesis within ChP epithelial cells into CSF; (ii) the transport of peripherally-derived proteins across the blood-CSF barrier and (iii) the clearance/reabsorption of proteins from CSF. There are established SILK methods for quantifying synthesis and clearance rates of Aβ, tau, APP,^14,15^ and here we report a novel method to determine the kinetics of proteins derived from, and reflective of the ChP function *in vitro* and *in vivo*. We found that the synthesis rate of TTHY was correlated with a direct measure of volume of CSF synthesized, suggesting this method could be an *in vivo* biomarker of CSF flow.

We used human ChP organoids to determine which proteins were most abundantly expressed by ChP epithelial cells, and which proteins were derived from organoid growth media and transported into the CSF-like fluid containing cysts (organoid CSF). Based on previous work using untargeted MS of organoid CSF and human CSF,^16^ paired with single-cell RNA sequencing to confirm which cells they derived from, we were able to establish that TTHY, CYTC and APOE were abundantly expressed by ChP epithelium and rapidly translated. Importantly, we found similar results in human CSF. We also confirmed that the only albumin found in organoid fluid was bovine and therefore derived from cell media rather than ChP organoids. It took much longer to appear in organoid fluid after labelling, consistent with albumin being transported across the ChP organoid rather than being produced by the ChP cells. Again, we found similar results *in vivo* for albumin and serotransferrin, indicating that they are likely produced peripherally by the liver and then transported across the blood-CSF barrier.

This isotope labelling method provides a novel tool that allows us to quantitate the synthetic function of the ChP *in vivo* and its capacity to transport proteins or drugs across the blood-CSF barrier. Since these findings are recapitulated in ChP organoids, with broadly similar synthesis (FSR) rates, this model has potential utility for understanding ChP physiology in a dish, and interrogate diseases of abnormal ChP physiology such as ChP tumours and idiopathic intracranial hypertension. ChP organoids may also have wider utility in testing factors which influence therapeutic drug delivery to the CNS.

We also aimed to develop a method capable of quantitating CSF protein clearance or resorption. Of the proteins identified, TTHY was of particular interest as it is produced mainly by the ChP,^26^ is rapidly translated and does not readily cross the blood-CSF barrier making it an attractive SILK target to monitor CSF clearance. Other proteins like APOE are known to be expressed by other cell types within the CNS.^27^ TTHY is a protein with a molecular mass of 55 kDa with a tetrameric structure. Physiologically it is involved in transporting the thyroid hormone thyroxine into the brain along with retinol-binding hormone.^28^ The two primary sites of TTHY synthesis are the liver,^29^ and the ChP,^30^ which give rise to TTHY in the plasma and CSF, respectively. However, there is an 11-fold difference in the mRNA levels and a 13-fold difference in the speed of synthesis between the two regions with faster production occurring in the ChP.^31^ Since rapid labelled leucine enrichment is seen both in organoids, where there is no other TTHY source, and *in vivo*, we conclude that most TTHY expression in CSF is derived from ChP. We next considered whether TTHY clearance was likely to be a good marker of CSF bulk flow clearance or whether it had other routes for removal. Unlike amyloid beta (Aβ), which may be cleared via the coordinated action of APOE and the low-density lipoprotein receptor-related protein 1 (LRP1),^32-34^ and possibly other transporters such as p-glycoprotein (PGP),^35^ removal of TTHY does not appear to be aided by transport proteins. To our knowledge, mechanisms of TTHY clearance from the brain have not been well studied. A limited number of studies have assessed the ability of TTHY to cross the blood-brain barrier (BBB) but have done so in the context of either TTHY-assisted Aβ transport,^36^ or TTHY- iododiflunisal (IDIF) transport.^37^ Both studies suggested that TTHY has the ability to cross the BBB but this likely only occurs in the brain-to-blood direction.^36^

To determine whether ChP protein kinetics, specifically TTHY, were also potential surrogate markers of CSF bulk flow, we investigated whether there was a relationship between TTHY FSR and the measured volume of CSF production captured using LiquoGuard. We found a strong correlation. We recognize that the movement of water and protein in the CSF may be partially independent of one another. Around 50% of water in the CSF compartment passes into this space by osmosis, and this process is driven by the osmotic draw of CSF proteins. Water can also be transported into the CSF space against an osmotic gradient,^38^ through active transport notably by the Na+/K+/2Cl− cotransporter (NKCC1) expressed in the luminal membrane of ChP cells.^39^ However, since it is practically difficult to label and track water *in vivo,* using a surrogate protein marker of ChP such as TTHY may be a valuable surrogate marker for tracking CSF flow *in vivo*. We propose that it should be tested in larger cohorts. The most striking difference between NPH and control subjects is the FCR (rate of clearance) of TTHY, which is ∼10 times lower in NPH. This was validated by monitoring a second TTHY peptide. A similar trend was seen for CYTC. We considered whether it could be an artefact of the site of CSF collection (controls had ventricular CSF sampling) or age of the controls, another potential confounding variable. We validated the results on a further independent control group of five cognitively normal, amyloid-negative, age-matched individuals from another institution (WashU) for whom isotopically labelled lumbar CSF was available. Since this cohort were labelled using the same protocol, were age-matched and had CSF collected in the same way, and from the same site, they represented an ‘ideal’ validation opportunity. This indicates that CSF protein turnover is ∼10 fold longer in NPH, suggesting possible fluid and protein stasis and greater potential for protein aggregation and/or post-translational modification.

Failed CSF clearance has become an important theme in NPH research, but the mechanism by which failed clearance of CSF occurs is yet to be established and there are currently no clinically available tools to capture this, other than imaging intrathecal contrast clearance, which is significantly delayed in NPH.^8^ Pathways of waste removal from the CNS has been a subject of investigation in recent years.^40^ The conventional view of brain fluid clearance and waste removal supports clearance via three routes; via arachnoid granulations into the dural venous sinuses; via nasal lymphatics and the cribriform plate into the cervical lymph nodes (CLNs) and via transporters/receptors located in the ChP epithelium.^41^ We do not yet know which biological process(es) lead to CSF protein clearance impairment in NPH, and this should be the direction of future studies.

We also observed significant differences in ChP protein translation, representing synthesis, in NPH compared with controls. The FSR of TTHY and serotransferrin were significantly lower in NPH providing *in vivo* evidence of reduced epithelial cell protein translation (TTHY) and reduced active transport across the ChP (serotransferrin). This may indicate that a feedback mechanism exists to regulate the ChP in humans.

Labelling ChP proteins using SILK could be a useful clinical diagnostic marker of NPH. Prolonged diagnostic lumbar drainage is currently the gold-standard diagnostic test to determine which individuals are likely to respond to CSF diversion,^42^ and requires a three-day hospital admission making it costly and burdensome for patients and healthcare providers. Labelling patients with a stable isotope prior to large volume CSF tap could provide objective evidence of clearance failure in addition to clinical evidence of gait improvement to provide a more accurate predictive test of clinical responders who might benefit from ventriculoperitoneal shunting, at a lower cost. This study was not designed to use ChP SILK to assess the clinical value of SILK to determine shunt response, and the majority (7/9) of individuals improved in response to CSF diversion, meaning we lacked a negative control group. However it provides confidence in participant selection, since most individuals demonstrated a sustained clinical benefit over years, and importantly most did not manifest an alternative neurodegenerative disease during the prolonged period of follow-up. To understand whether these findings are specific to NPH, and whether they are correlated with clinical symptoms, larger studies that include symptomatic and asymptomatic patients with DESH, as well as other forms of congenital hydrocephalus will be valuable.

There is also likely to be considerable interest in a kinetic protein biomarker that reflects CSF protein turnover in other areas of neuroscience. Notably in intrathecal therapeutics where compounds such as antisense oligonucleotides (ASO) are administered directly into CSF. The rate at which an individual circulates CSF and CSF protein is likely to influence drug delivery, clinical efficacy and drug safety. Multiple ASO studies have been terminated early owing to safety concerns. The Htt ASO trialled in Huntington's disease was terminated in part due to ventriculomegaly.^43^ This could have been a manifestation of accelerated neurodegeneration with accelerated volume loss. The other explanation is that they developed stasis of CSF circulation caused by increased CSF protein concentration or inflammation.

This study has some limitations. A major challenge in studying NPH is the lack of diagnostic biomarkers. We selected cases that had clinical features that met international criteria for probable NPH,^18^ and radiological support for disproportionately enlarged subarachnoid-space hydrocephalus for NPH. All individuals were examined by a board-certified neurologist prior to inclusion to screen for other more likely diagnoses (e.g. PSP). We were also able to establish that 5/5 cases tested had reversibility of ventricular volume following ventriculoperitoneal shunting, which is strongly associated with clinical reversibility.^44^ We were also able to clinically follow individuals for years to demonstrate sustained clinical improvement consistent with NPH, and to confirm they did not develop another neurodegenerative disease. Two had *ex vivo* brain pathology.

We acknowledge that there are inevitable sources of confound which we were unable to fully account for in this proof of concept study. Ventricular volumes of individuals with NPH are significantly higher than controls,^45^ but still represent no more than 50% total CSF volume and unlikely to explain the 10-fold difference in clearance rates observed in this study. Nor is this likely to explain why the kinetics of specific proteins are altered, and others not. We could not control for this variable since we did not have access to ventricular volumes for all participants. We also acknowledge some of limitations of measuring CSF flow using a liquoGuard machine, which could alter blood flow to the brain,^46^ or fluid shifts between physiological compartments. A strength of the study is to have validated findings in two independent control cohorts, equivalent results in ventricular and lumbar CSF and in two different centres, supporting previous unpublished data that SILK is reproducible between clinical centres.

In conclusion, our SILK method provides a potentially valuable tool for quantifying ChP function and CSF protein dynamics *in vivo* and *in vitro*. ChP organoids recapitulate human ChP cells, clarify which proteins are being expressed by the ChP and highlight the utility of this model in studying diseases of CSF disruption. The identification of TTHY as a potential kinetic biomarker of CSF flow *in vivo* offers a novel approach for diagnosing and monitoring NPH.

## Supplementary Material

fcag029_Supplementary_Data

## Data Availability

Data available from the corresponding author on reasonable request.
