# Supplementary material for: CSF protein clearance impairment revealed using stable isotope kinetics in normal pressure hydrocephalus
Source: Brain Commun. 2026 Feb 4;8(1):fcag029. doi: 10.1093/braincomms/fcag029 (PMC12926509; doi:10.1093/braincomms/fcag029)
Supplement: fcag029_Supplementary_Data [file fcag029_supplementary_data.pdf]

# Supplementary Materials for

## CSF clearance impairment captured using stable isotope labelling kinetics (SILK) in normal pressure hydrocephalus

**Table of contents**

**Supplementary Figure 1.** Kinetic measurements in choroid plexus organoid CSF (iCSF).

**Supplementary Figure 2.** Choroid plexus protein kinetics in ChP organoid lysates.

**Supplementary Figure 3.** Summary of NPH imaging.

**Supplementary Figure 4.** Comparison of protein kinetics between five lumbar CSF control subjects.

**Supplementary Table 1.** Demographic and biomarker data for NPH and control cohorts.

**Supplementary Table 2.** Summary of ChP peptides, FSR and FCR in NPH and control cohorts.

**Supplementary Table 3.** ChP SILK peptides and ion transitions in the multiplexed ChP assay.

**Graphical Abstract License**

This supplementary material has been provided by the authors to give readers additional information about their work.

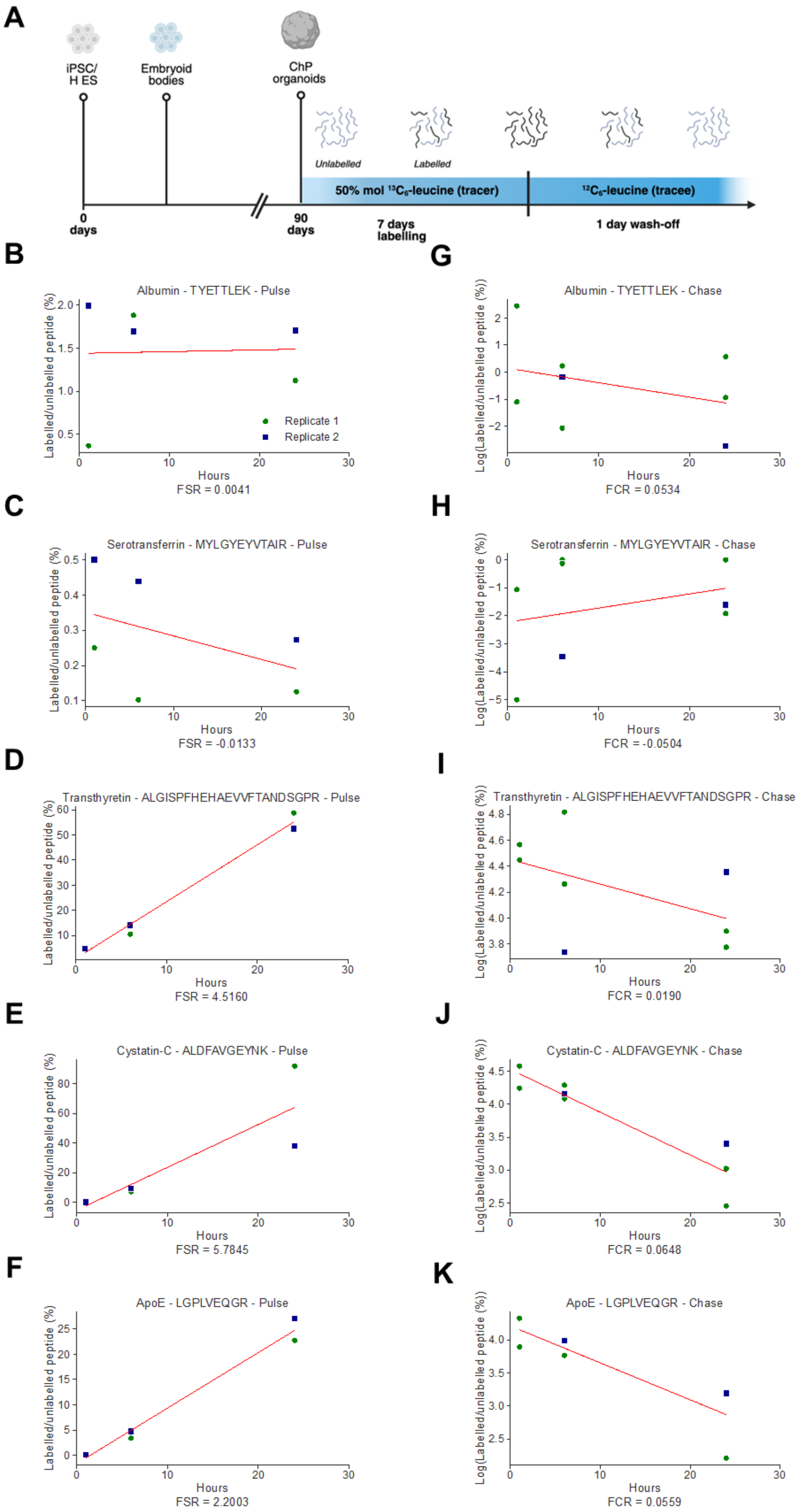
A
iPSC/ H ES
Embryoid bodies
ChP organoids
Unlabelled
Labelled
50% mol $^{13}C_6$-leucine (tracer)
$^{12}C_6$-leucine (tracee)
0 days
90 days
7 days labelling
1 day wash-off
B
Albumin - TYETTLEK - Pulse
Labelled/unlabelled peptide (%)
Replicate 1
Replicate 2
Hours
FSR = 0.0041
C
Serotransferrin - MYLGYEYVTAIR - Pulse
Labelled/unlabelled peptide (%)
Hours
FSR = -0.0133
D
Transthyretin - ALGISPFHEHAEVVFTANDSGPR - Pulse
Labelled/unlabelled peptide (%)
Hours
FSR = 4.5160
E
Cystatin-C - ALDFAVGEYNK - Pulse
Labelled/unlabelled peptide (%)
Hours
FSR = 5.7845
F
ApoE - LGPLVEQGR - Pulse
Labelled/unlabelled peptide (%)
Hours
FSR = 2.2003
G
Albumin - TYETTLEK - Chase
Log(Labelled/unlabelled peptide (%))
Hours
FCR = 0.0534
H
Serotransferrin - MYLGYEYVTAIR - Chase
Log(Labelled/unlabelled peptide (%))
Hours
FCR = -0.0504
I
Transthyretin - ALGISPFHEHAEVVFTANDSGPR - Chase
Log(Labelled/unlabelled peptide (%))
Hours
FCR = 0.0190
J
Cystatin-C - ALDFAVGEYNK - Chase
Log(Labelled/unlabelled peptide (%))
Hours
FCR = 0.0648
K
ApoE - LGPLVEQGR - Chase
Log(Labelled/unlabelled peptide (%))
Hours
FCR = 0.0559

**Supplementary Figure 1 Kinetic measurements in choroid plexus organoid CSF (iCSF).** (**A**) Schematic showing development and $^{13}C_6$-leucine labelling of ChP organoids. (**B-F**) Fractional synthesis rates of plasma and choroid plexus derived proteins in iCSF: (**B**) Albumin. (**C**) Serotransferrin. (**D**) TTHY. (**E**) Cys-C. (**F**) ApoE. (**G-K**) Fractional clearance rates of plasma and ChP derived proteins in iCSF: (**G**) Albumin. (**H**) Serotransferrin. (**I**) TTHY. (**J**) Cys-C. (**K**) ApoE. The proteotypic peptides used for each protein are indicated. Dark blue squares and green circles represent two independent organoid inductions, (*n* = 2). Red lines represent the simple linear regression model used for FSR/FCR calculations (please see Material and Methods). Abbreviations: ApoE = Apolipoprotein E; ChP = Choroid Plexus; CSF = cerebrospinal fluid; Cys-C = Cystatin-C; FCR = Fractional Clearance Rate; FSR = Fractional Synthesis Rate; TTHY = Transthyretin. Fig. S2A created in BioRender. Murphy, E. (2025) https://BioRender.com/fz42kxc

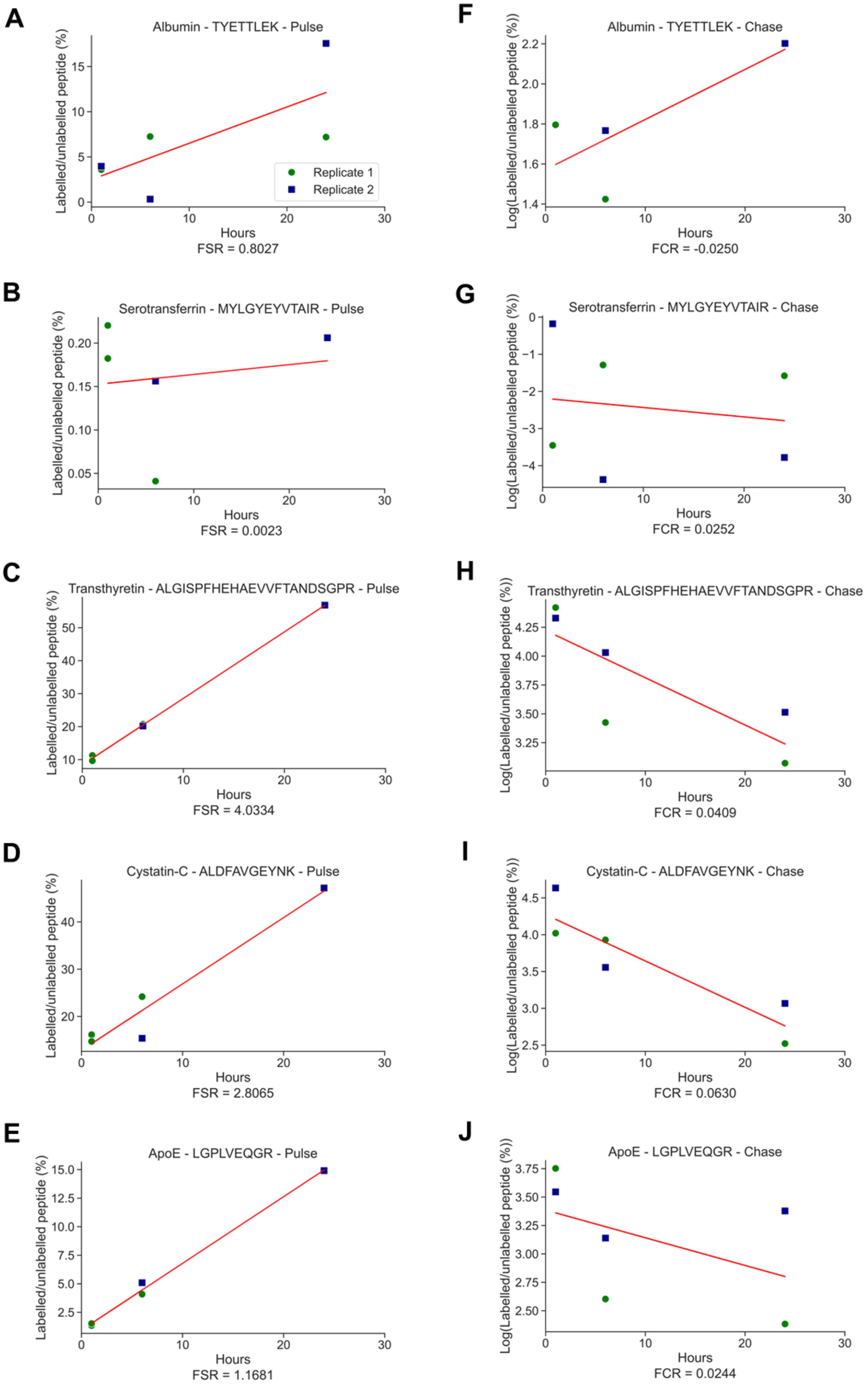
A
Albumin - TYETTLEK - Pulse
Labelled/unlabelled peptide (%)
Replicate 1
Replicate 2
Hours
FSR = 0.8027
B
Serotransferrin - MYLGYEYVTAIR - Pulse
Labelled/unlabelled peptide (%)
Hours
FSR = 0.0023
C
Transthyretin - ALGISPFHEHAEVVFTANDSGPR - Pulse
Labelled/unlabelled peptide (%)
Hours
FSR = 4.0334
D
Cystatin-C - ALDFAVGEYNK - Pulse
Labelled/unlabelled peptide (%)
Hours
FSR = 2.8065
E
ApoE - LGPLVEQGR - Pulse
Labelled/unlabelled peptide (%)
Hours
FSR = 1.1681
F
Albumin - TYETTLEK - Chase
Log(Labelled/unlabelled peptide (%))
Hours
FCR = -0.0250
G
Serotransferrin - MYLGYEYVTAIR - Chase
Log(Labelled/unlabelled peptide (%))
Hours
FCR = 0.0252
H
Transthyretin - ALGISPFHEHAEVVFTANDSGPR - Chase
Log(Labelled/unlabelled peptide (%))
Hours
FCR = 0.0409
I
Cystatin-C - ALDFAVGEYNK - Chase
Log(Labelled/unlabelled peptide (%))
Hours
FCR = 0.0630
J
ApoE - LGPLVEQGR - Chase
Log(Labelled/unlabelled peptide (%))
Hours
FCR = 0.0244

**Supplementary Figure 2 Choroid plexus protein kinetics in ChP organoid lysates.** (**A-E**) Fractional synthesis rates of plasma and ChP derived proteins in ChP organoid lysate: (**A**) Albumin. (**B**) Serotransferrin. (**C**) TTHY. (**D**) Cys-C. (**E**) ApoE. (**F-J**) Fractional clearance rates of plasma and ChP derived proteins in ChP organoid lysate: (**F**) Albumin. (**G**) Serotransferrin. (**H**) TTHY. (**I**) Cys-C. (**J**) ApoE. The proteotypic peptides used for each protein are indicated. Dark blue squares and green circles represent two independent organoid inductions, ($n$ = 2). Red lines represent the simple linear regression model used for FSR/FCR calculations (please see Material and Methods). Abbreviations: ApoE = Apolipoprotein E; ChP = Choroid Plexus; Cys-C = Cystatin-C; FCR = Fractional Clearance Rate; FSR = Fractional Synthesis Rate; TTHY = Transthyretin.

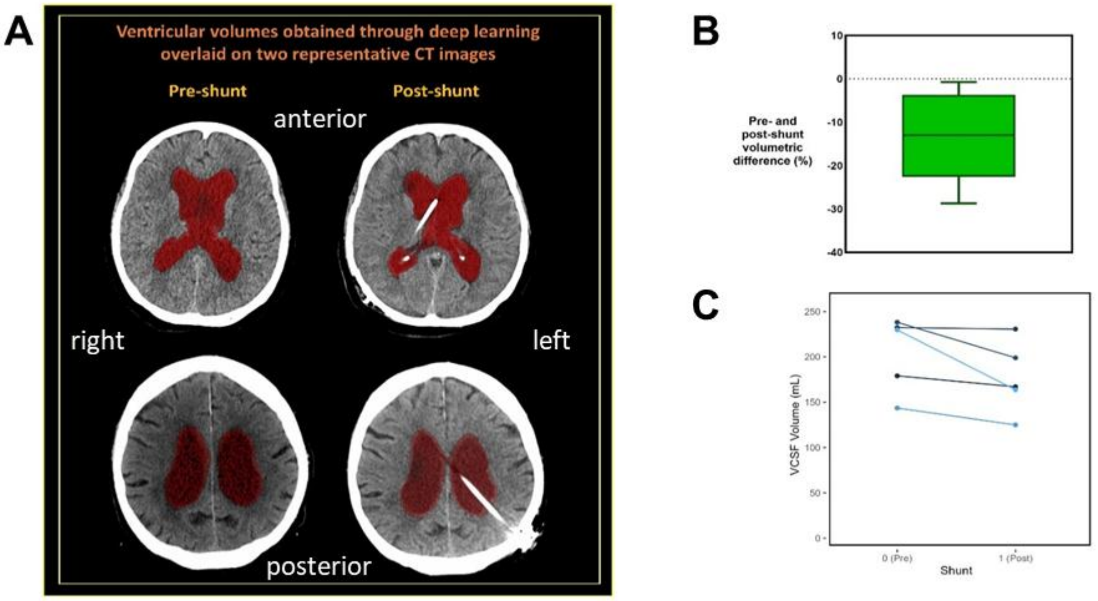


**Supplementary Figure 3 Summary of NPH imaging.** (**A**) VCSF segmentation outputs obtained using deep-learning-derived CT brain quantification pipeline in two representative pre- and post-shunt paired CT datasets. (**B**) VCSF volume changes (in percentage, %) between pre- and post-shunt VCSF images (*n* = 10 pre- and post-shunt CT scans from five unique participants from the NPH cohort underwent brain segmentation analysis). (**C**) VCSF volumes in millilitres before and after shunt. (*n* = 10 pre- and post-shunt CT scans from five unique participants from the NPH cohort underwent brain segmentation analysis). Abbreviations: CSF = cerebrospinal fluid; VCSF = Ventricular CSF.

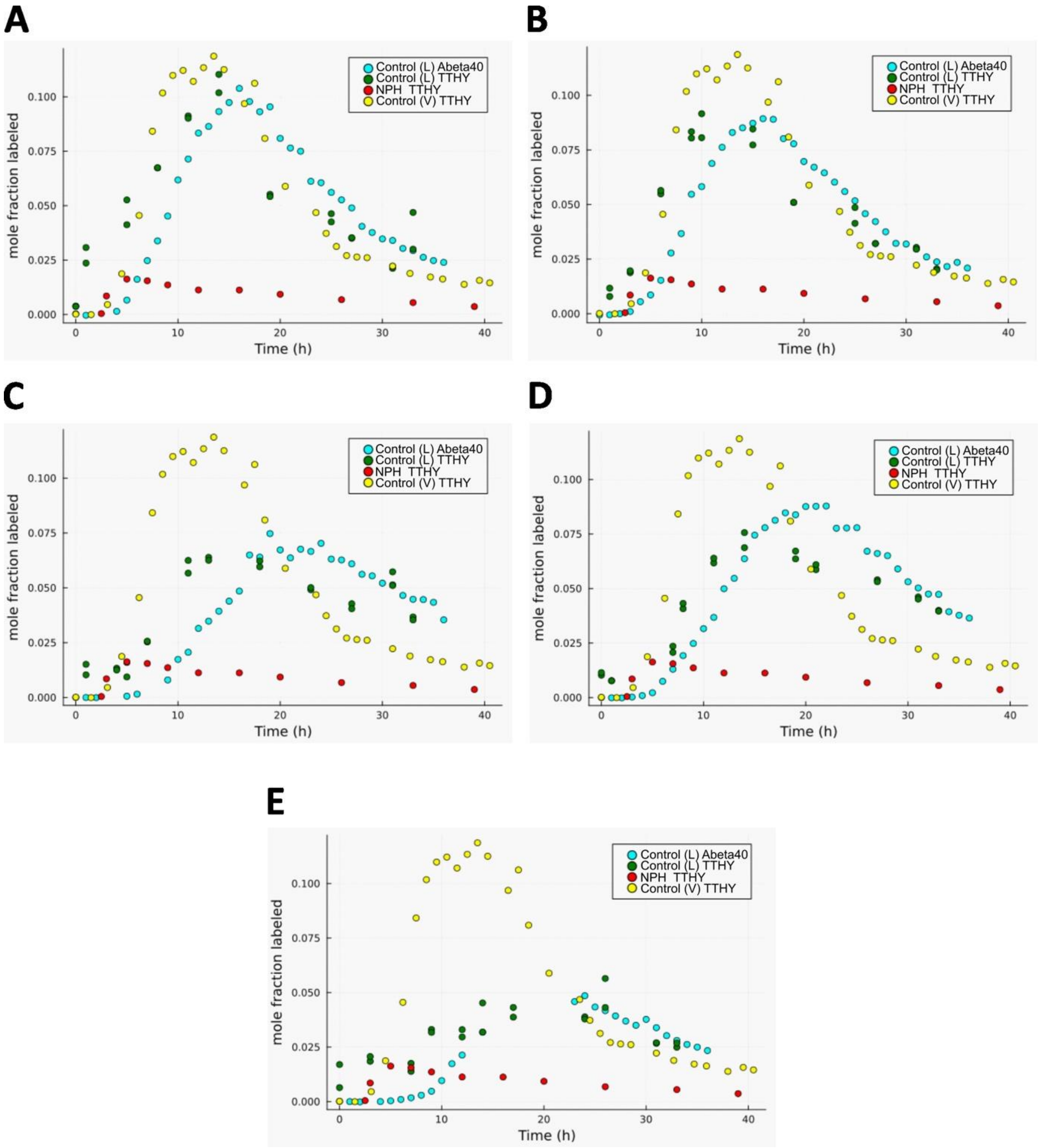


**Supplementary Figure 4 Comparison of protein kinetics between five lumbar CSF control subjects.** (**A-E**) Kinetics of amyloid beta 40 peptide (Abeta40) and transthyretin (TTHY) of each subject. Each dot represents the measured mole fraction labelled at a given timepoint. This is a measure of the amount of labelled peptide over the total peptide pool. For aiding comparisons, the average turnover of TTHY in the NPH- and pSAH (ventricular CSF) cohorts are shown. Abbreviations: Abeta = amyloid beta 40 peptide; CSF = cerebrospinal fluid; L = Lumbar; NPH = Normal Pressure Hydrocephalus; TTHY = Transthyretin; V = Ventricular.

**Supplementary Table 1 | Demographic and biomarker data for NPH and control cohorts.**

| | NPH<br>(*n* = 10) | Controls (post SAH)<br>(*n* = 4) | Controls (validation)<br>(*n* = 5) |
|---|---|---|---|
| Age at LP (years) | 75 (71-78) | 57 (46-65) | 70 (63-84) |
| % Male | 80 | 0 | 40 |
| % Caucasian | 90 | NA | 80 |
| MMSE | 28 (26-28)<br>(*n* = 9) | NA | NA |
| Baseline 10m walk time (seconds) | 11 (10-23)<br>(*n* = 9) | NA | NA |
| Number responding to shunting | 7 | NA | NA |
| % Executive Dysfunction | 50 | NA | NA |
| % Episodic memory problems | 70 | NA | NA |
| % Language Impairment | 20 | NA | NA |
| % Gait disturbance | 100 | NA | NA |
| % Parkinsonism | 20 | NA | NA |
| % Positive for Falls | 44<br>(*n* = 9) | NA | NA |
| % Urinary Incontinence | 80 | NA | NA |
| % Cerebellar signs | 11<br>(*n* = 9) | NA | NA |
| % Eye movement abnormalities | 33<br>(*n* = 9) | NA | NA |
| % Supranuclear gaze palsy | 13<br>(*n* = 8) | NA | NA |
| % Pyramidal signs | 11<br>(*n* = 9) | NA | NA |
| CSF production rate (ml/hr) | 84 (62-89) | NA<br>NA | |
| Clinical CSF Aβ42/40 | 0.118 (0.082-0.129) | | |

| | ($n$ = 9) |
|---|---|

Median and interquartile ranges are shown

(Interquartile ranges are reported to the nearest whole number)

Where data was missing, the number of subjects for which the data were available is indicated within parentheses

LP = Lumbar Puncture; MMSE = Mini-Mental State Examination; NPH = Normal Pressure Hydrocephalus; NA = Not Available.

**Supplementary Table 2 | Summary of ChP peptides, FSR and FCR in NPH and control cohorts.**

| Protein | Peptide Sequence | Domain | Amino Acids | FSR (%/hr) | | Sig. | FCR (%/hr) | | Sig. |
|---|---|---|---|---|---|---|---|---|---|
| | | | | *NPH* | *Control (V)* | | *NPH* | *Control (V)* | |
| Transthyretin | ALGISPFHEHAEVVFTANDSGPR (abbreviated as 'ALG') | Transthyretin/ hydroxyisourate hydrolase | 101- 123 | 2.29 (1.27-3.78) n=8 | 10.79 (9.81-34.10) n=3 | p < 0.05 (0.012) | 0.74 (0.02-1.16) n=8 | 7.25 (2.28-11.19) n=4 | p < 0.005 (0.004) |
| | TSESGELHGLTTEEEFVEGIYK (abbreviated as 'TSE') | | 69-90 | 1.64 (0.94-2.64) n=9 | 8.64 (8.36-26.35) n=3 | p < 0.01 (0.009) | 1.57 (1.05-2.08) n=8 | 9.45 (5.76-13.79) n=4 | p < 0.005 (0.004) |
| Cystatin-C | ALDFAVGEYNK | N/A | 52-62 | 0.55 (0.17-1.24) n=9 | 4.16 (2.23-4.53) n=3 | p < 0.01 (0.009 | 2.00 (0.74-2.60) n=6 | 3.45 (2.54-4.36) n=2 | NS (0.143) |
| Serotransferrin | MYLGYEYVTAIR | Transferrin-like 1 | 332-343 | 0.05 (0.03-0.09) n=9 | 0.47 (0.27-0.48) n=3 | p < 0.01 (0.009) | *Not captured* | *Not captured* | |
| Albumin | TYETTLEK | Albumin 2 | 376-383 | 0.03 (0.01-0.03) n=8 | 0.10 (0.06-0.12) n=3 | p < 0.05 (0.012) | *Not captured* | *Not captured* | |
| Apolipoprotein E | LQAEAFQAR | Lipid-binding and lipoprotein association region | 270-278 | 0.35 (0.08-0.84) n=8 | 1.77 (1.11-2.00) n=3 | p < 0.05 (0.012) | 1.40 (0.39-7.58) n=7 | 1.14 (n=1) | NS (0.827) |
| ***Exploratory analysis between NPH and the Control (L) validation cohort*** | | | | | | | | | |
| | | | | *NPH* | *Control (L)* | | *NPH* | *Control (L)* | |
| Transthyretin | ALGISPFHEHAEVVFTANDSGPR ('ALG') | Transthyretin/ hydroxyisourate hydrolase | 101-123 | 2.29 (1.27-3.78) n=8 | 4.35 (2.00-7.00) n=5 | NS (0.093) | 0.74 (0.02-1.16) n=8 | 4.51 (3.39-6.14) n=5 | p < 0.005 (0.002) |
| | TSESGELHGLTTEEEFVEGIYK ('TSE') | | 69-90 | 1.64 (0.94-2.64) n=9 | 3.95 (0.94-5.03) n=5 | NS (0.060) | 1.57 (1.05-2.08) n=8 | 6.22 (2.97-8.50) n=5 | p < 0.005 (0.004) |

FSR (%/hr) and FCR (%/hr) data represented as group median (min-max values)
NS = Not Significant
*Not captured* = not long enough chase period to capture clearance curve (only production curve observed)
Sig. (*P*-values; exact significance) calculated by Mann Whitney U test
Control (V) = SAH-Control Cohort (Montpellier); Control (L) = Validation lumbar CSF Control Cohort (Washington University in St Louis/Montpellier).

**Supplementary Table 3 | ChP SILK peptides and ion transitions in the multiplexed ChP assay.**

| Protein | Peptide Sequence | Amino Acids | Precursor ion (m/z) | Precursor charge (z) | Product ion (type) | Product ion (m/z) |
|---|---|---|---|---|---|---|
| Albumin | TYETTLEK | 376-383 | 492.7478 | 2 | y6+ | 720.3774 |
| | | | | | y5+ | 591.3348 |
| | TYETT**L[$^{13}$C$_6$]**EK | | 495.7579 | 2 | y6+ | 726.3975 |
| | | | | | y5+ | 597.3549 |
| Apolipoprotein E | LQAEAFQAR | 270-278 | 517.2749 | 2 | b3+ | 313.1870 |
| | | | | | y3+ | 374.2146 |
| | **L[$^{13}$C$_6$]**QAEAFQAR | | 520.2850 | 2 | b3+ | 313.1870 |
| | | | | | y3+ | 374.2146 |
| Cystatin-C | ALDFAVGEYNK | 52-62 | 613.8062 | 2 | y5+ | 610.2831 |
| | | | | | b6+ | 617.3293 |
| | A**L[$^{13}$C$_6$]** DFAGEYNK | | 616.8163 | 2 | y5+ | 610.2831 |
| | | | | | b6+ | 623.3495 |
| Serotransferrin | MYLGYEYVTAIR | 332-343 | 739.8710 | 2 | y9+ | 1071.5469 |
| | | | | | y7+ | 851.4621 |
| | MY**L[$^{13}$C$_6$]** GYEYVTAIR | | 742.8811 | 2 | y9+ | 1071.5469 |
| | | | | | y7+ | 851.4621 |
| Transthyretin | ALGISPFHEHAEVVFTANDSGPR | 101-203 | 613.5567 | 4 | y6+ | 645.2951 |
| | | | | | y4+ | 416.2252 |
| | A**L[$^{13}$C$_6$]**GISPFHEHAEVVFTANDSGPR | | 615.0618 | 4 | y6+ | 645.2951 |
| | | | | | y4+ | 416.2252 |
| | TSESGELHGLTTEEEFVEGIYK | 69-90 | 819.0552 | 3 | y4+ | 480.2817 |
| | | | | | y5+ | 609.3243 |
| | TSESGE**L[$^{13}$C$_6$]**HGLTTEEEFVEGIYK | | 821.0619 | 3 | y4+ | 480.2817 |
| | | | | | y5+ | 609.3243 |
| | TSESGE**L[$^{13}$C$_6$]**HG**L[$^{13}$C$_6$]**TTEEEFVEGIYK | | 823.0686 | 3 | y4+ | 480.2817 |
| | | | | | y5+ | 609.3243 |
| **Internal standard** | | | | | | |
| Yeast enolase | LGANAILGVSLAASR | 106-120 | 706.9146 | 2 | y8+ | 760.4312 |
| | | | | | y11+ | 1057.6364 |
| | GNPTVEVELTTEK | 16-28 | 708.8645 | 2 | y11++ | 623.3323 |
| | | | | | y8+ | 948.4884 |

*SILK peptide transitions include heavy isotope labelled leucine and are shown by inclusion of* ***L[$^{13}$C$_6$]*** *in the above peptide sequences.*
*Both quantitative and qualitative product ions are listed, with the quantitative ion for each peptide/precursor ion stated first.*

49 Spadina Ave. Suite 200
Toronto ON M5V 2J1 Canada
www.biorender.com

# Confirmation of Publication and Licensing Rights - Open Access

**September 15th, 2025**

***Subscription Type:*** *Institution - Academic*
***Agreement number:*** *FF28RC6569*
***Publisher Name:*** *Oxford Academic*

***Figure Title:*** *Graphical Abstract ChP-SILK*

***Citation to Use:*** *Created in BioRender. Alvarez Giovannucci, T. (2025) https://BioRender.com/833qp0x*

To whom this may concern,

This document ("Confirmation") hereby confirms that Science Suite Inc. dba BioRender ("BioRender") has granted the following BioRender user: Tatiana Alvarez Giovannucci ("User") a BioRender Academic Publication License in accordance with BioRender's Terms of Service and Academic License Terms ("License Terms") to permit such User to do the following on the condition that all requirements in this Confirmation are met:

1) publish their Completed Graphics created in the BioRender Services containing both User Content and BioRender Content (as both are defined in the License Terms) in publications (journals, textbooks, websites, etc.); and
2) sublicense such Completed Graphics under "open access" publication sublicensing models such as CC-BY 4.0 and more restrictive models, so long as the conditions set forth herein are fully met.

Requirements of User:

1) All Completed Graphics to be published in any publication (journals, textbooks, websites, etc.) must be accompanied by the following citation either as a caption, footnote or reference for each figure that includes a Completed Graphic:
"Created in BioRender. Alvarez Giovannucci, T. (2025) https://BioRender.com/833qp0x ".
2) All terms of the License Terms including all Prohibited Uses are fully complied with. E.g. For Academic License Users, no commercial uses (beyond publication in journals, textbooks or websites) are permitted without obtaining or switching to a BioRender Industry Plan.
3) A Reader (defined below) may request that the User allow their figure to be a public template for Readers to view, copy, and modify the figure. It is up to the User to determine what level of access to grant.

Open-Access Journal Readers:

Open-Access journal readers ("Reader") who wish to view and/or re-use a particular Completed Graphic in an Open-Access journal subject to CC-BY sublicensing may do so by clicking on the URL link in the applicable citation for the subject Completed Graphic.

The re-use/modification options below are available after the Reader requests the User to adapt their figure as a BioRender template and the User has granted such access.

1) View-Only/Free Plan Use: A Reader who wishes to only view the Completed Graphic may do so in the BioRender Services as either a BioRender Free Plan user or simply as a viewer. By becoming a BioRender Free Plan user, the Reader may view, modify and re-use the Completed Graphic as permitted under BioRender's Basic License Terms (e.g. personal use only, no publishing or commercial use permitted).
2) Re-Use/Publish with No Modifications: For any re-use and re-publication of a Completed Graphic with no modification(s) to the Completed Graphic made by the Reader, a Reader may do so by citing the original author using the citation noted above with the Completed Graphic. The Reader must also comply with the underlying License Terms which apply to the Completed Graphic as noted above (e.g. no commercial use for Academic License).
3) Re-Use/Publish with Modifications: For any re-use and re-publication of a Completed Graphic with a modification(s) made by the Reader, the Reader may do so by becoming a BioRender user themselves under either an Academic or Industry Plan, citing the original author using the citation noted above with the Completed Graphic and complying with the applicable License Terms.

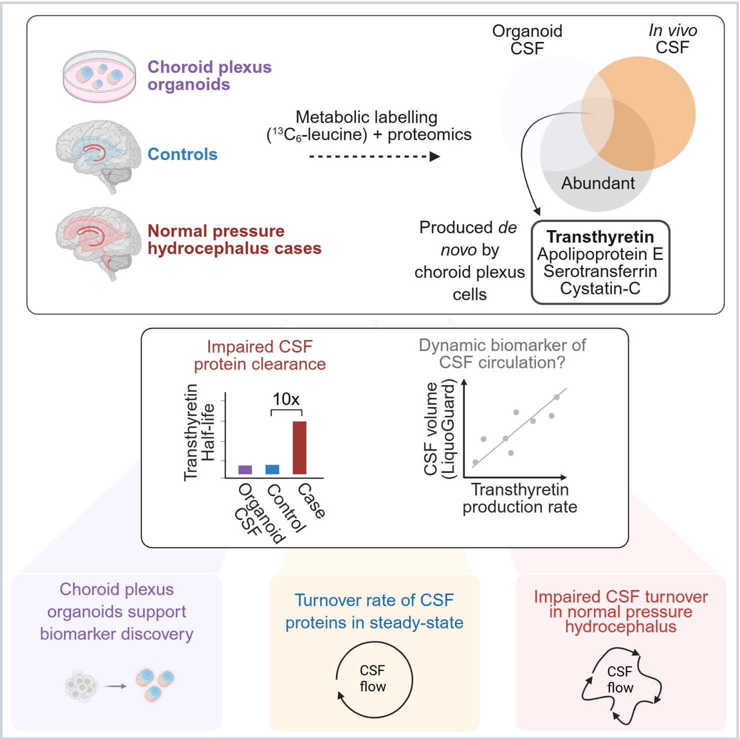


*For any questions regarding this document, or other questions about publishing with BioRender, please refer to our BioRender Publication Guide, or contact BioRender Support at support@biorender.com.*
